# Toward a Combination of Biomarkers for Molecular Characterization of Multiple Sclerosis

**DOI:** 10.3390/ijms232214000

**Published:** 2022-11-13

**Authors:** Dafni Birmpili, Imane Charmarke Askar, Lucas Dinh Pham-Van, Thomas Kuntzel, Caroline Spenlé, Aurélien Riou, Dominique Bagnard

**Affiliations:** CNRS UMRS7242, Biotechnology and Cell Signaling, Therapeutic Peptides Team, Institut du Médicament de Strasbourg (IMS), ESBS, 300 Boulevard S. Brant, 67400 Illkirch-Graffenstaden, France

**Keywords:** multiple sclerosis, biomarkers, experimental autoimmune encephalomyelitis, RNA

## Abstract

Multiple sclerosis (MS) is an autoimmune disease affecting the central nervous system associated with chronic inflammation, demyelination, and axonal damage. MS is a highly heterogeneous disease that leads to discrepancies regarding the clinical appearance, progression, and therapy response of patients. Therefore, there is a strong unmet need for clinically relevant biomarkers capable of recapitulating the features of the disease. Experimental autoimmune encephalomyelitis (EAE) is a valuable model for studying the pathophysiology of MS as it recapitulates the main hallmarks of the disease: inflammation, blood-brain barrier (BBB) disruption, gliosis, myelin damage, and repair mechanisms. In this study, we used the EAE-PLP animal model and established a molecular RNA signature for each phase of the disease (onset, peak, remission). We compared variances of expression of known biomarkers by RT-qPCR in the brain and spinal cord of sham and EAE animals monitoring each of the five hallmarks of the disease. Using magnetic cell isolation technology, we isolated microglia and oligodendrocytes of mice of each category, and we compared the RNA expression variations. We identify genes deregulated during a restricted time frame, and we provide insight into the timing and interrelationships of pathological disease processes at the organ and cell levels.

## 1. Introduction

Multiple sclerosis (MS) is a chronic autoimmune disease affecting the central nervous system (CNS). It is an inflammatory and neurodegenerative disease characterized by demyelinating lesions and axonal damage [1]. MS is a complex disease characterized by important heterogeneity in radiological and histopathological features, thus affecting the clinical appearance, progression, and therapeutic response of each patient [2,3]. This heterogeneity is probably due to the location of lesions and the underlying biological mechanisms [4]. Despite the major progress achieved in understanding biological mechanisms within this heterogeneous disease, the exact pathophysiological process of MS remains elusive. In fact, little is known about the sequence, timing, and interrelationships between the different pathological processes that lead to disease. It is, therefore, of utmost importance to define the time frame for the deregulation of specific biomarkers that will reflect the different features of the disease. This will deepen our understanding of MS and facilitate the diagnosis, prediction of clinical outcome, and therapeutic choice among the growing number of current treatments available for MS.

Experimental autoimmune encephalomyelitis (EAE) is the most commonly used experimental model for the study of MS as it resembles the pathological features of human MS in many aspects, including inflammation, demyelination, axonal loss, gliosis, and immune reaction [5]. Studies in EAE have allowed the elucidation of pathogenetic pathways and the development of all approved MS therapies, which further validates the suitable correlation between EAE models and MS [6,7].

In this study, we used the EAE-PLP animal model. PLP (proteolipid protein) is the major protein constituent of CNS myelin. Immunization of mice of a susceptible strain, such as SJL/J mice with PLP_139-151,_ initiates expansion and differentiation of PLP-specific autoimmune T lymphocytes that result in a chronic disease initiated by a first paralytic episode followed by multiple remissions and relapses, thus mimicking the relapsing-remitting course of human MS [5,8]. In fact, this model is characterized by three main disease phases: onset, peak, and remission. In this work, in order to establish a molecular signature for each phase of the disease, we monitored the variances of gene expression of known biomarkers at different stages of the disease. We divided these biomarkers into six distinct categories that reflect the major pathological hallmarks leading to the common symptoms of EAE: inflammation, blood-brain barrier (BBB) disruption, gliosis, oligodendrocyte and neuronal damage, microglial activation and finally, repair mechanisms. We focused on RNA biomarkers, studied by qPCR, that are easily quantifiable and dynamically change in relation to the ongoing pathological alterations. We performed a time-course analysis of 23 known MS biomarkers to monitor the molecular changes occurring during each disease phase and obtain a global dynamic transcriptomic signature. We chose clinically relevant, well-known MS molecular biomarkers that recapitulate and sensitively monitor early changes in the aforementioned MS pathological stages. The selected biomarkers have been used individually in mouse models of EAE and showed suitable translatability into clinical relevance. A comprehensive review of the chosen biomarkers can be found in Birmpili et al., 2022 [9].

We show strong and reproducible expression profiles for a panel of genes that sensitively monitor the hallmarks of MS, thereby identifying sets of deregulated genes usable to characterize the disease phases at the molecular level in a time-dependent manner.

## 2. Results

### 2.1. EAE Molecular Signature in the Spinal Cord and Brain

#### 2.1.1. Inflammation-Related Genes

**Figure 1:**
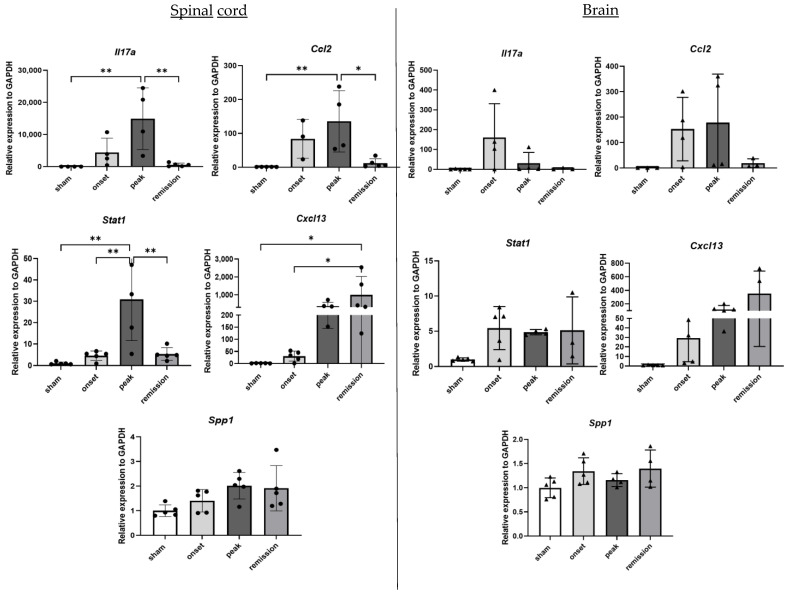
Differential expression of genes representative of inflammation in CNS organs during the different phases of PLP-induced EAE. The levels of mRNA transcripts for the selected genes relative to *Gapdh* were measured in the spinal cord (**left**) and brain (**right**) samples isolated from adjuvant controls and EAE mice on the onset, peak, and remission phases by RT-qPCR. A number of 3 to 5 mice was used in the experiment. Each symbol represents a sample. * *p* < 0.05, ** *p* < 0.01 one-way ANOVA.

In order to evaluate the kinetics of inflammation during EAE, the gene expression of the cytokines IL17A, CCL2, STAT1, the chemokine CXCL13 and the extracellular matrix (ECM) protein osteopontin (coded by the *Spp1* gene) were quantified by RT-qPCR in spinal cord and brain samples at different time points. Generally, inflammation-related genes are, as expected, strongly upregulated. *Il17a*, *Ccl2* and *Stat1* RNA levels show a gradual increase from the onset until the peak of the disease, followed by a massive decrease and return to basal levels during remission. Conversely, the strong expression of *Cxcl13* persists until the remission phase. Finally, *Spp1* seems to slightly increase only during the peak of the disease in the spinal cord samples although its levels did not reach statistical significance (Figure 1).

#### 2.1.2. BBB Disruption-Related Genes

BBB breakdown is an early step of MS. It is characterized by an altered impermeability of endothelial cells tight junctions and transendothelial migration of immune cells in the CNS. Among the molecular factors involved in this complex process, matrix metalloproteinases (MMP) play a crucial role by modulating the function of tight junctions worsening the inflammation, and facilitating lymphocyte passage [10,11].

**Figure 2:**
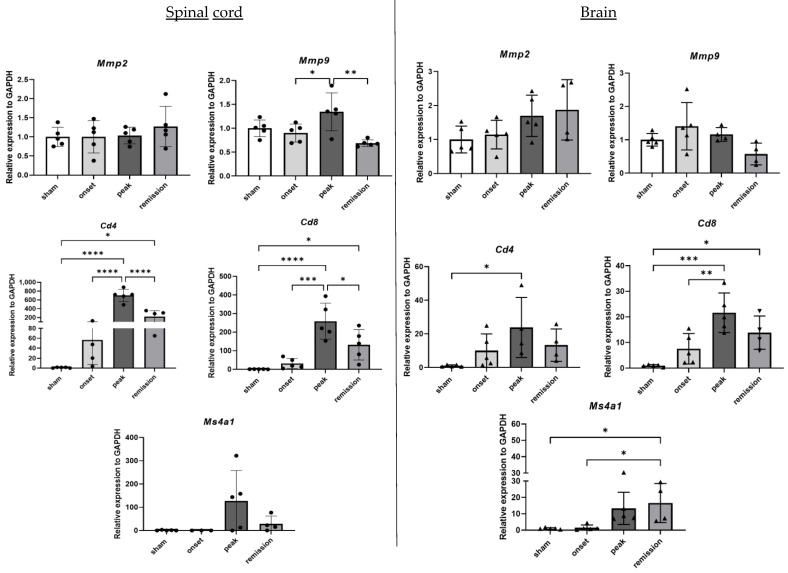
Differential expression of genes representative of BBB disruption in CNS organs during the different phases of PLP-induced EAE. The levels of mRNA transcripts for the selected genes relative to *Gapdh* were measured in the spinal cord (**left**) and brain (**right**) samples isolated from adjuvant controls and EAE mice on the onset, peak, and remission phases by RT-qPCR. A number of 3 to 5 mice was used in the experiment. Each symbol represents a sample. * *p* < 0.05, ** *p* < 0.01, *** *p* < 0.001, **** *p* < 0.0001, one-way ANOVA.

While not exclusive of BBB disruption, the expression of MMP2 and MMP9 were measured as a readout of the BBB leakage. This was strengthened by the measure of T and B-cell markers as an indirect readout of BBB disruption followed by lymphocyte infiltration. Our results show that there is no statistical difference in the expression of the metalloproteinase MMP2. A slight increase in the mRNA levels of *Mmp9* during the acute phase is observed, followed by a decrease during remission. On the other hand, a gradual upregulation is observed for the genes illustrating the infiltrating immune cells, T lymphocytes CD4 and CD8, during the onset and peak phases. Their expression diminishes during remission. A late increase in the expression of the B-cell differentiation antigen CD20 (*Ms4a1*) is observed only during the peak phase. The deregulation amplitude of immune cell markers is less important in the brain samples reflecting less lesion load and cellular infiltration (Figure 2).

#### 2.1.3. Astrocyte-Related Genes

**Figure 3:**
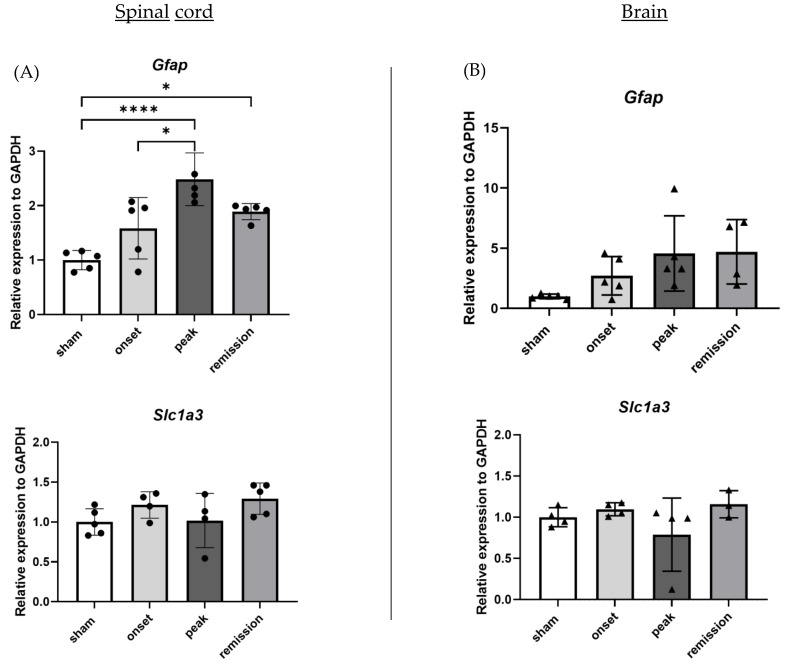
Differential expression of genes representative of astrogliosis in CNS organs during the different phases of PLP-induced EAE. The levels of mRNA transcripts for the selected genes relative to *Gapdh* were measured in the spinal cord (**A**) and brain (**B**) samples isolated from adjuvant controls and EAE mice on the onset, peak, and remission phases by RT-qPCR. A number of 3 to 5 mice was used in the experiment. Each symbol represents a sample. * *p* < 0.05, **** *p* < 0.0001, one-way ANOVA.

Gliosis was studied using GFAP and GLAST (coded by the gene *Slc1a3*). Interestingly, even though gene expression of *Slc1a3* levels did not differ between the groups, *Gfap* expression is particularly increased in the EAE condition in the spinal cord samples. Its expression increases gradually until the peak of the disease, and its levels remain elevated even during the remission phase for both brain and spinal cord samples (Figure 3).

#### 2.1.4. Oligodendrocyte and Neuronal Damage-Related Genes

**Figure 4:**
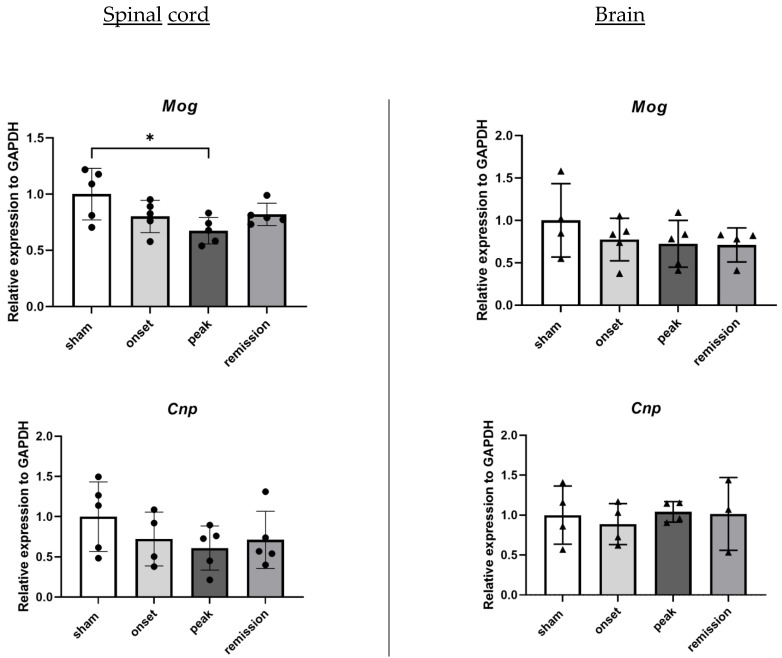
Differential expression of genes representative of oligodendrocyte damage in CNS organs during the different phases of PLP-induced EAE. The levels of mRNA transcripts for the selected genes relative to *Gapdh* were measured in the spinal cord (**left**) and brain (**right**) samples isolated from adjuvant controls and EAE mice on the onset, peak, and remission phases by RT-qPCR. A number of 3 to 5 mice was used in the experiment. Each symbol represents a sample. * *p* < 0.05 one-way ANOVA.

**Figure 5:**
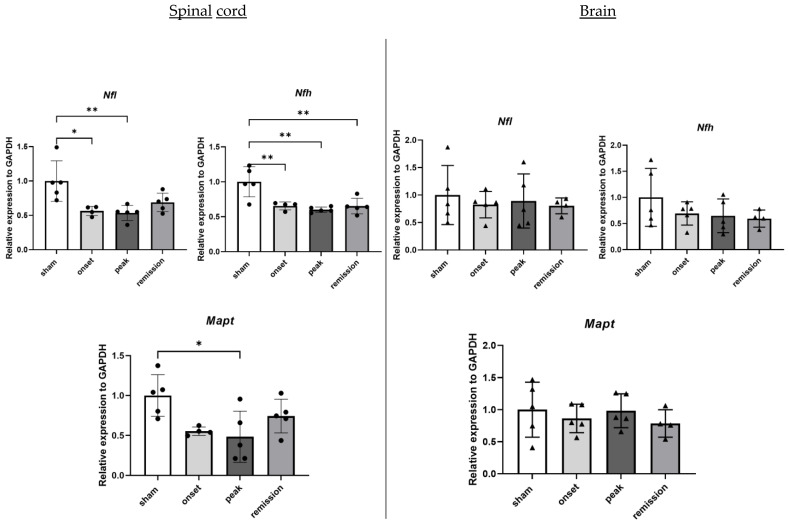
Differential expression of genes representative of neuronal damage in CNS organs during the different phases of PLP-induced EAE. The levels of mRNA transcripts for the selected genes relative to *Gapdh* were measured in the spinal cord (**left**) and brain (**right**) samples isolated from adjuvant controls and EAE mice on the onset, peak, and remission phases by RT-qPCR. A number of 3 to 5 mice was used in the experiment. Each symbol represents a sample. * *p* < 0.05, ** *p* < 0.01, one-way ANOVA.

Although the RNA levels of *Cnp* do not differ significantly between the groups, the attacks on mature oligodendrocytes are reflected by the decrease in *Mog* RNA levels in the spinal cord samples at the peak of the disease coinciding with the maximal degree of disability (Figure 4). Neuronal damages are reflected by the important decrease in neurofilament (*Nfh* and *Nfl*) and *Mapt* RNA levels during the onset and peak of the disease that still persist during remission (Figure 5).

#### 2.1.5. Microglia Activation-Related Genes

To elucidate the activation state of microglia, we used TMEM119 as a specific microglial cell-surface marker [12]. CHI3L1 and Urokinase receptor (uPAR) were used as microglia activation markers [13,14].

**Figure 6:**
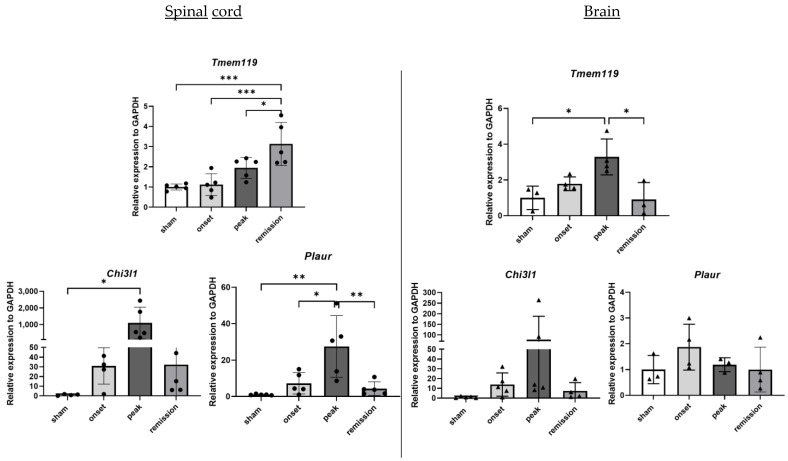
Differential expression of genes representative of microglial activation in CNS organs during the different phases of PLP-induced EAE. The levels of mRNA transcripts for the selected genes relative to *Gapdh* were measured in the spinal cord (**left**) and brain (**right**) samples isolated from adjuvant controls and EAE mice on the onset, peak, and remission phases by RT-qPCR. A number of 3 to 5 mice was used in the experiment. Each symbol represents a sample. * *p* < 0.05, ** *p* < 0.01, *** *p* < 0.001, one-way ANOVA.

Interestingly, we can see that *Tmem119* expression slowly increases, reaching its maximum level during the remission phase for the spinal cord samples. Interestingly in the brain, we can see a different pattern of deregulation. *Tmem119* levels reach their maximum at the peak phase and then return to a basal state at the remission phase. The expression of activation markers is massively increased during the onset and peak phases, with *Chi3l1* reaching an upregulation of more than 1,000-fold. Their expression is significantly decreased during remission (Figure 6).

#### 2.1.6. Repair Mechanisms Related Genes

**Figure 7:**
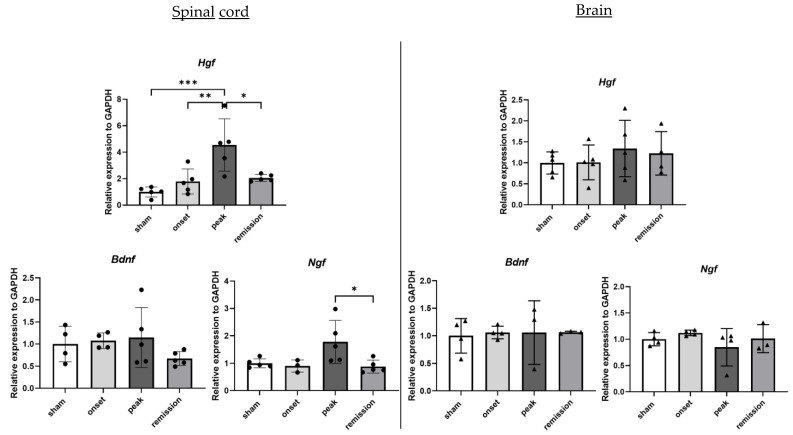
Differential expression of genes representative of repair mechanisms in CNS organs during the different phases of PLP-induced EAE. The levels of mRNA transcripts for the selected genes relative to *Gapdh* were measured in the spinal cord (**left**) and brain (**right**) samples isolated from adjuvant controls and EAE mice on the onset, peak, and remission phases by RT-qPCR. A number of 3 to 5 mice was used in the experiment. Each symbol represents a sample. * *p* < 0.05, ** *p* < 0.01, *** *p* < 0.001, one-way ANOVA.

In this work, to study the regenerative capacities of the CNS, we opted for neurotrophic factors, including neurotrophins and growth factors. We opted for the neurotrophic markers BDNF, NGF and the growth factor HGF. Surprisingly, there is no notable difference in the mRNA expression levels of the neurotrophins *Bdnf* and *Ngf* during the different EAE phases. On the other hand, *Hgf* expression is gradually increased in the EAE condition in the spinal cord samples (Figure 7).

**Figure 8:**
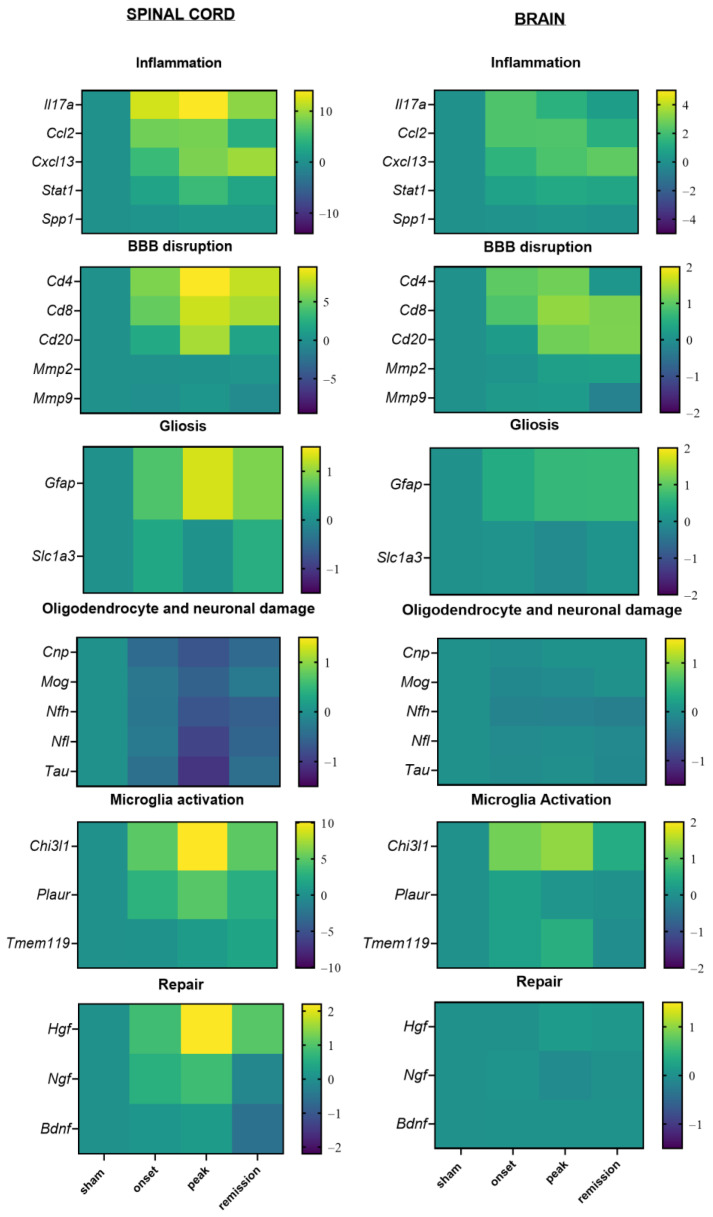
Heatmap representing the differential expression of genes in the spinal cord (**left**) and brain (**right**) during the different phases of PLP-induced EAE. The levels of mRNA transcripts for the selected genes were measured in spinal cord and brain samples isolated from EAE mice at the onset, peak, and remission phase and were compared to adjuvant controls (sham). A number of 3 to 5 mice was used in the experiment (logarithmic scale bars).

Our findings reveal a distinct pattern in gene expression alterations in the CNS organs and especially in the spinal cord, during the early stages of PLP-induced EAE with the upregulation of inflammation-related genes. During the peak phase, the upregulation of astrogliosis and microglial activation markers coincides with the downregulation of oligodendrocyte and neuronal-associated markers. During the remission phase, the upregulation of *Tmem119* and *Gfap* indicates that despite clinical amelioration, glial cell activation is still present and important. Surprisingly, the neurotrophic markers show no alteration in the RNA level in our study (Figure 8).

### 2.2. EAE Molecular Signature in Cells Type

#### 2.2.1. Oligodendrocyte-Specific Changes in Gene Expression

**Figure 9:**
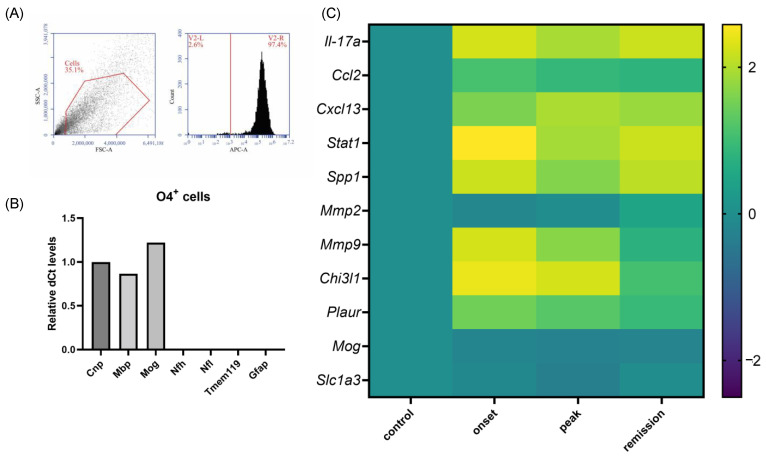
Validation of purity and differential expression in O4^+^ sorted cells during the different phases of PLP-induced EAE. (**A**) Flow cytometry analysis validating the purity of the obtained O4 cell population. (**B**) The levels of mRNA transcripts for the selected genes representative of other CNS cells were measured in O4^+^ cell samples isolated from naive and EAE mice on the onset, peak, and remission phases by RT-qPCR. (**C**) The levels of mRNA transcripts for the selected genes relative to *Gapdh* were measured in O4^+^ cell samples isolated from naive and EAE mice on the onset, peak, and remission phases by RT-qPCR. A total of 3 mice were used in the experiment (logarithmic scale bar).

O4^+^ cells were isolated from the whole brains of naive and EAE mice at different time points of the disease. O4 expression begins in late oligodendrocyte progenitors. Consequently, our oligodendrocyte samples include late progenitors, pre-myelinating, and myelinating oligodendrocytes. The cells were isolated from adult brains dissociated with a combination of physical and enzymatic dissociation, density gradient separation, and magnetic bead sorting. The purity of the collection was verified by flow cytometry for the expression of the oligodendrocyte marker O4 (Figure 9A) and RT-qPCR at the mRNA level for a set of oligodendrocyte markers (*Cnp*, *Mog,* and *Mbp*) being all highly expressed while glial (*Tmem119*, *Gfap)* and neuronal markers (*Nfl* and *Nfh*) were not detectable (Figure 9B).

We performed qPCR analysis for the same panel of genes as for the organs. We show only the detectable genes (Ct < 35). Our analysis shows an important upregulation of inflammatory markers in the oligodendrocytes derived from EAE mice. Less distinction was observed between the profiles of oligodendrocytes derived from naive and remission tissues. Most of the detected gene deregulation occurred at the onset phase of the disease (Figure 9C and Supplementary Figure S1). The top differentially expressed genes for the onset phase are *Stat1* (379-fold, *p* = 0.0009), *Mmp9* (177-fold, *p* = 0.0315), *Il17a* (177-fold), *Chi3l1* (268-fold), and *Spp1* (154-fold). Their expression levels remained elevated during the peak phase, although their upregulation is less important. The expression of *Mog* was, as expected, downregulated (2-fold, *p* = 0.0371) following the autoimmune attack on mature oligodendrocytes.

#### 2.2.2. Specific Gene Expression Changes in CNS Phagocytic Cells

**Figure 10:**
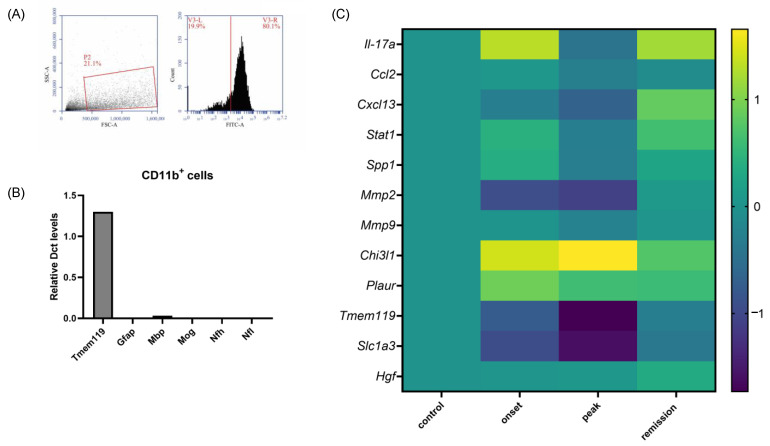
Validation of purity and differential expression in CD11b^+^ sorted cells during the different phases of PLP-induced EAE. (**A**) Flow cytometry analysis validating the purity of the obtained CD11b cell population. (**B**) The levels of mRNA transcripts for the selected genes representative of other CNS cells were measured in CD11b^+^ cell samples isolated from naive and EAE mice on the onset, peak, and remission phases by RT-qPCR. (**C**) The levels of mRNA transcripts for the selected genes relative to *Gapdh* were measured in CD11b^+^ cell samples isolated from naive and EAE mice on the onset, peak, and remission phases by RT-qPCR. A total of 2–3 mice were used in the experiment (logarithmic scale).

To identify the transcriptomic changes occurring in the microglia related to the EAE model pathology, we extracted CD11b^+^ cells from the brains of naive and EAE animals at the different stages of the disease. CD11b is an antigen expressed in microglia and macrophages; hence, our samples include both cell types in the EAE condition. However, since macrophages are rare in healthy tissue, microglia constitute the major cell type in the naive condition. The purity of the collection was verified by flow cytometry using a CD11b antibody (Figure 3A). The purity of the obtained cell population was further verified by RT-qPCR, demonstrating the high expression of *Tmem119* (microglial marker) and the low expression of genes associated with other glial cells (*Mog*, *Mbp*, *Gfap*) and neurons (*Nfl* and *Nfh*) (Figure 3B). The top differentially upregulated genes for the onset phase are *Chi3l1* (25-fold), *Il17a* (20.3-fold, *p* = 0.0433), and *Plaur* (8-fold, *p* = 0.0399). Another significantly deregulated marker is *Spp1* (osteopontin, 2.3-fold, *p* = 0.0167). *Chi3l1* expression was significantly upregulated at the peak phase (45-fold, *p* = 0.0420) and *Tmem119* expression was highly downregulated (50-fold) (Figure 10 and Supplementary Figure S2).

## 3. Discussion

The aim of this study was to investigate the mRNA expression profile of different factors involved in the major physiopathological hallmarks of MS during the three distinct disease phases of the EAE-PLP model. Spinal cord and brain samples were collected at the onset, peak, and remission phases and were then examined by qPCR targeting different biomarkers. We divided these biomarkers into six categories that reflect the major pathological hallmarks of MS: inflammation, blood-brain barrier disruption, gliosis, demyelination followed by neurodegeneration, microglial activation and repair mechanisms. These events are well known but how they are related and when exactly they are initiated remains unclear. We used the EAE model to study the timing and interconnections of these pathological hallmarks during the three distinct phases of the EAE-PLP model to establish molecular signatures reflecting the whole pathological process. EAE is a valuable tool for the study of MS immunopathology because it recapitulates all the main features of the disease and has proved to be efficient in biomarker screening. None of the selected genes showed significant deregulation simultaneously during the three phases.

Our results indicate that inflammation coincides with EAE severity, with the cytokines IL17A, STAT1, and CCL2 being dramatically overexpressed during the onset and peak of the disease. BBB leakage was indirectly represented in our model by the upregulation of markers of lymphocytes (CD4, CD8) in the CNS. The fact that the expression profile of CD4 and CD8 remains upregulated during the remission phase reflects ongoing immune cell infiltration at this stage, implying a continuous permeability of the BBB. Our results show significant but less immune infiltration in the brain, highlighting the fact that the spinal cord presents a more important lesion load. The levels of metalloproteinases, known to play a major role in BBB disruption, were unaltered in the mRNA level when measured at the whole organ level. However, MMPs are regulated in several levels: gene transcription, synthesis, secretion as an inactive proenzyme, activation, potential inhibition by their inhibitor (TIMP), and glycosylation, which protects MMPs from degradation. This could mean that during MS, MMPs are not necessarily upregulated at the transcriptional level. Furthermore, it is important to note that MMPs are not only implicated in BBB disruption but also in inflammation. In fact, they generate chemokine gradients that lead to inflammatory cell recruitment and amplify inflammatory responses [15].

From our analysis in both the organ and cellular levels, two leading targets are revealed: *Chi3l1* and *Spp1*.

CHI3L1 is a chitinase-like glycoprotein mainly expressed by microglia, macrophages, and reactive astrocytes in areas of active demyelination [16]. Its function in the CNS is not clear and appears to be dual. On the one hand, CHI3L1 levels in the CSF are predictive of conversion from Clinically Isolated Syndrome (CIS) to definite RRMS [17]. Plasma levels are increased in patients with a progressive disease compared to a relapsing-remitting [18]. On the other hand, it seems to also have protective effects as in vitro experiments showed that it can induce oligodendrogenesis in neural stem cells [19] and CHI3L1-deficient mice show more severe EAE [20]. In our model, we can observe a dramatic increase in the mRNA levels of *Chi3l1* in both the organ and cellular levels. Its expression is increased in the spinal cord, microglia, and oligodendrocytes of EAE mice from the onset to the peak phase. In both cases, its expression returns to basal levels during the remission phase. Our results showing an upregulation of *Chi3l1* in EAE microglia are consistent with two studies showing that microglia from MS patients express more CHI3L1 compared to microglia derived from control patients [21,22]. However, to our knowledge, this is the first time that a study has found that CHI3L1 is expressed by oligodendrocytes derived from EAE mice. In fact, CHI3L1 is not expressed at all in the naive oligodendrocyte population (Ct > 40), but we observe a gradual increase in its expression with a massive expression in the oligodendrocytes of the peak condition (Ct ~ 28–30). While the exact role function of CHI3L1 in oligodendrocytes remains to be elucidated, it is tempting to speculate that oligodendrocytes express CHI3L1 as a defense mechanism following the autoimmune attack.

The second interesting target revealed by our profiling is *Spp1* coding for osteopontin. OPN is a pleiotropic phosphoprotein functioning whether as a free cytokine in body fluids or as an extracellular matrix molecule implicated in inflammation and tissue remodeling. It is produced by immune cells such as T cells and macrophages, and glial cells as reactive astrocytes and microglia [23]. Transcriptomic studies in MS lesions have shown an abundant expression of OPN transcripts, which are completely absent in the healthy brain [24]. Studies in the EAE-MOG model have shown that mice deficient for OPN show milder clinical scores with downregulation of proinflammatory cytokines [24]. Our analysis of the whole spinal cord and brain samples failed to reveal *Spp1* as a major target. Its upregulation is revealed only in the sorted populations of oligodendrocytes and microglia during the onset phase. This is in concordance with a study by Selvaraju et al. on the cuprizone model [25]. This study revealed that microglia in demyelinating brain regions expressed osteopontin. The study by Masuda et al. has found an increase in osteopontin levels in MS patients derived microglia [22]. However, this study being a single-cell analysis focusing on microglia, macrophages were excluded from the analysis.

As seen for *Chi3l1*, *Spp1* expression is not detectable in naive oligodendrocytes, but in oligodendrocytes derived from onset EAE mice, its expression is increased. Studies have shown that *Spp1* stimulates the proliferation and differentiation of NG-2 glial cells into oligodendrocytes [26]. Its maximum increase during the onset, followed by gradual reduction during the peak phase, could be a mechanism of defense that gradually shuts down, finally leading to remyelination impairment.

Furthermore, the transcriptomic analysis in the CNS organs of EAE mice highlighted the importance of microglia in the pathology with strong upregulation of *Chi3l1* and *Plaur,* both markers expressed (between others) by activated microglia [20] and *Tmem119*, representative of microglial numbers [27].

When compared to other CNS cells or tissue macrophages, microglia have a distinct transcriptome signature [21]. During the EAE immune attacks, the most infiltrating cells are macrophages. One of the markers allowing the differentiation between microglia and tissue-derived macrophages is TMEM119, expressed only in microglia [27]. Interestingly, in our model, we observed the downregulation of this marker during the EAE phases. This downregulation could be explained by the fact that our sorted CD11b^+^ population includes both microglia and macrophages. Macrophages being absent in the naive brain but also being the major immune infiltrate in the EAE CNS means that our population is highly enriched by macrophages not expressing TMEM119. However, studies have described a downregulation of the TMEM119 marker in relation to neuropathological diseases in animal models without macrophage infiltration, such as the cuprizone model, where there is no BBB disruption. This result has also been validated at the histological level in MS lesions [22]. So, our result could represent not only a change in the composition of the obtained cellular fraction but also an inherent downregulation of this gene.

Microglia adopt a variety of different morphologies and are distributed unevenly in the brain [28]. In fact, transcriptionally and functionally different subpopulations of microglia exist even in the naive brain. In MS tissue also, there are heterogenous populations of microglia. Microglia adopt a phenotype between an anti-inflammatory state and a proinflammatory phenotype, which can have at the same time detrimental and protective effects in MS [29]. Our work presents a limitation because it cannot distinguish the different microglial populations and differentiate them from macrophages. Furthermore, in our study, to isolate microglia using an extracellular marker, we opted for CD11b. CD11b, also named integrin subunit alpha M (ITGAM), is expressed on the surface of many leukocytes, including monocytes, neutrophils, macrophages, and microglia. However, in the CNS of the EAE model, the vast majority of CD11b^+^ cells are microglia and macrophages. To overcome this limitation, a single-cell or spatial transcriptomics analysis could be interesting.

In any case, our results show that microglia/macrophages derived from EAE brains are able to rapidly change their transcriptomic profile presenting a time-dependent molecular signature. The signatures obtained from the single-cell analysis of Masuda et al. showed that human “homeostatic” microglia profiles have distinct expression patterns and partially overlap with those of adult mice [22]. This reinforces our idea that the differences that we obtain in our model could be representative of MS disease.

Concerning our oligodendrocyte samples, we can observe a strong upregulation of immunity-related genes such as STAT1, IL17A, and CCL2. Oligodendrocytes are known to express IL17A [30] and CCL2 [31] but, to our knowledge, this is the first time that an increased and persistent expression of inflammation related genes is observed during the EAE phases. These observations correlate with the increasing evidence replacing oligodendrocytes as active players in MS and not only passive target cells by the autoimmune attacks [32,33].

In summary, our results show that the study of mRNA expression profiles in the CNS of mice at different phases of the EAE gives us insight into the timing and sequence of the deregulation of the pathological hallmarks of the disease. The analysis at the cellular level reveals information that can be diluted in the whole organ analysis. Our analysis revealed *Chi3l1* and *Spp1* as interesting targets massively deregulated in our model that could be interesting biomarkers. Our analysis on the cellular level highlights the importance of microglia in the EAE condition and the implication of oligodendrocytes in the immune pathways. We believe that the transcriptomic analysis in the CNS of EAE mice can be used as a valuable tool for the discovery of biomarkers and for the evaluation of the efficiency of treatments for MS.

## 4. Materials and Methods

### 4.1. Animals

SJL/J female mice were obtained from Janvier Labs. Mice were bred and kept with a 12 h light-dark cycle, with free access to food and water under specific pathogen-free conditions. The study was conducted following the European Commission directive 2010/63/EU for the protection of animals used for scientific purposes and approved by the regional animal care committee under the French ministry of agriculture agreement APAFIS#22687-2019102813393158. Mice were allowed to acclimatize to the animal house facility for at least one week before EAE induction. The Hooke EAE-PLP induction kit was used according to the manufacturer’s instructions (EK-2120, Hooke laboratories, Lawrence, MA, USA). Briefly, at 8–9 weeks old, mice were anesthetized with isoflurane and immunized subcutaneously with 0.1 mg of Myelin Proteolipid protein (PLP_139-151_) peptide emulsified in complete Freund’s adjuvant (CFA) and intraperitoneally with 0.1 mg of Pertussis toxin (PTX). Controls included a sham group, which received all the injections, including CFA and PTX but not the PLP_135-151_ peptide. All mouse groups (sham and EAE) were subjected to the same handling and were weighed and scored daily from the 7th day post-immunization to assess disease severity. Clinical score was assessed according to the following criteria: 0, no disease; 1, tail plegia; 2, impaired righting reflex and partial hind limb paresis; 3, complete hind limb paralysis; 4, hind limb paralysis with partial forelimb paralysis; and 5, moribund or dead. The first clinical signs of disability (onset phase) appeared approximately 9 days after immunization. The onset phase corresponds to a score of 0.5, reflecting tail paresis. The acute phase appeared around day 13, when the symptoms reached their peak with a maximum score of 3, reflecting complete paralysis of the two hind limbs. Then a remission phase was observed around day 15.

### 4.2. Tissue Isolation

Mice were euthanized by intraperitoneal injection of a lethal dose of a ketamine-xylazine solution. Then, mice were transcardially perfused with ice-cold PBS to wash out the blood capillaries. Spinal cords and brains were resected, snap-frozen in liquid nitrogen, and stored at −80 °C until further use.

### 4.3. Cell Separation

Firstly, brains were dissociated into single-cell suspensions using the adult brain dissociation kit from Miltenyi (Bergisch Gladbach, Germany) (#130-107-677) based on a combination of enzymatic and mechanical dissociation protocol. Briefly, tissues are sliced into 8 sagittal sections and placed in tubes containing an enzyme cocktail. Each sample was then dissociated for 30 min at 37 °C on the gentleMACS Octo Dissociator with Heaters (#130-096-427, Miltenyi Biotec) using the 37C_ABDK_01 program. Then, myelin and debris were removed with Debris Removal solution (#130-109-398, Miltenyi Biotec). The resulting cell suspension was labeled with a CD11b antibody (#130-093-634) from Miltenyi or an O4 antibody (#130-096-670) and placed on a MACS^®^ column placed in the magnetic field of a MACS separator. CD11b^+^ cells were eluted, and their purity was validated by flow cytometry using the CD11b-FITC antibody (#130-113-796) or an O4-APC antibody (#130-115-810) from Miltenyi and the BD AccuriTM Cytometer. Cells were resuspended in Trizol and kept at −80 °C until further use.

### 4.4. RNA Extraction and Quantitative Real-Time PCR

Total RNA was isolated from spinal cords and brains using TRI Reagent (#T9424, Merck, Darmstadt, Germany) according to the manufacturer’s instructions. Briefly, the organs were homogenized by using ceramic beads and the Minilys tissue homogenizer (Bertin instruments, Montigny-le-Bretonneux, France). The RNA extraction was continued with a classic phenol-chloroform extraction. The RNA quality was assessed with the Agilent 2100 bioanalyzer electrophoresis system. Only samples with a RIN (RNA integrity number) superior to 7.5 were retained for further analysis.

After DNase treatment (Roche, Basel, Switzerland), cDNA was synthesized from 500 ng total RNA using the High-Capacity cDNA Reverse Transcription Kit (Applied Biosystems, Waltham, MA, USA), and qPCR was performed using the Biorad (Hercules, CA, USA) CFX Connect Real-Time System with Taqman Real-time PCR Master Mix (#10370395, Applied Biosystems, Waltham, MA, USA). The Taqman probes are listed in Supplementary Table S1. *Gapdh* was used as a housekeeping gene. The relative gene expression ratio was calculated based on the 2^−ΔΔCT^ method.

### 4.5. Statistical Analysis

All statistical analyses were performed with Graphpad Prism 9. For quantitative RT-qPCR analyses, a one-way ANOVA test followed by Tukey’s multiple comparison test was used to identify significant changes in gene expression between the different disease phases of EAE. Data are presented as mean ± standard deviation, and the *p* values < 0.05 were considered statistically significant.

## Data Availability

Raw data can be available on demand.

## Supplementary Materials

The following supporting information can be downloaded at: https://www.mdpi.com/article/10.3390/ijms232214000/s1, Figure S1: Differential expression of genes in O4^+^ cells during the different phases of PLP-induced EAE. The levels of mRNA transcripts for the selected genes relative to Gapdh were measured in O4^+^ samples isolated from control and EAE mice on the onset, peak and remission phase by RTqPCR. A number of 3 mice was used in the experiment. Each symbol represents a sample. * *p* < 0.05, ** *p* < 0.01, *** *p* < 0.001, one-way ANOVA.; Figure S2: Differential expression of genes in CD11b^+^ cells during the different phases of PLP-induced EAE. The levels of mRNA transcripts for the selected genes relative to Gapdh were measured in CD11b^+^ samples isolated from controls and EAE mice on the onset, peak and remission phase by RT-qPCR. A number of 2 to 3 mice was used in the experiment. Each symbol represents a sample. * *p* < 0.05, ** *p* < 0.01, *** *p* < 0.001, one-way ANOVA.; Table S1: List of probes used for qPCR.
