# Supplementary material for: Toward a Combination of Biomarkers for Molecular Characterization of Multiple Sclerosis
Source: Int J Mol Sci. 2022 Nov 13;23(22):14000. doi: 10.3390/ijms232214000 (PMC9695566; doi:10.3390/ijms232214000)
Supplement: Supplementary file 1 [file ijms-23-14000-s001.zip › ijms-1933935-supplementary.pdf]

Supplementary Materials

Supplementary Table S1. List of probes used for qPCR

Gene name	Probe reference
Bdnf	Mm01334043 m1
Ccl2	Mm00441242 m1
Cd20	Mm00545909 m1
Cd4	Mm00442754 m1
Cd8	Mm01182107 g1
Ch3l1	Mm00657889 mH
Cxcl13	Mm01208154 g1
Gfap	Mm01253033 m1
Hgf	Mm01135184 m1
Il17a	Mm00439616 m1
Mbp	Mm01266402 m1
Mmp2	Mm00439498 m1
Mmp9	Mm0442991 m1
Mog	Mm01279062 m1
Nfh	Mm01191456 m1
Nfl	Mm01315666 m1
Ngf	Mm0443039 m1
Spp1	Mm00436767 m1
Stat1	Mm00439518 m1
Mapt	Mm00521988 m1
Plaur	Mm01149438 m1
Slc1a3	Mm00600697 m1
Tmem119	Mm005255305 m1
Gapdh	Mm999999915 g1

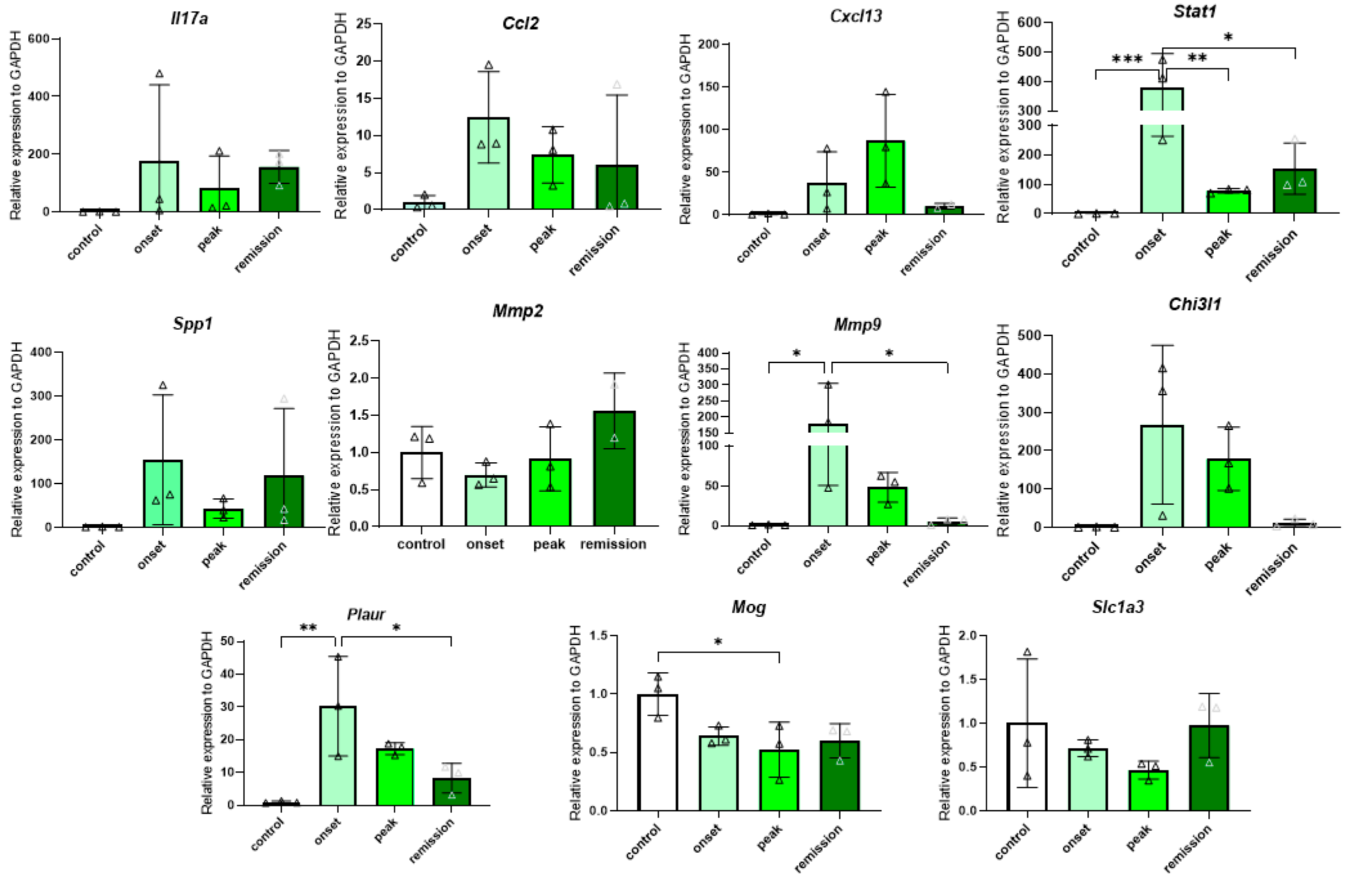

Supplementary Figure S1. Differential expression of genes in O4+ cells during the different phases of PLP-induced EAE. The levels of mRNA transcripts for the selected genes relative to Gapdh were measured in O4+ samples isolated from control and EAE mice on the onset, peak and remission phase by RTqPCR. A number of 3 mice was used in the experiment Each symbol represents a sample. * $p < 0.05$, ** $p < 0.01$, *** $p < 0.001$, one-way ANOVA.

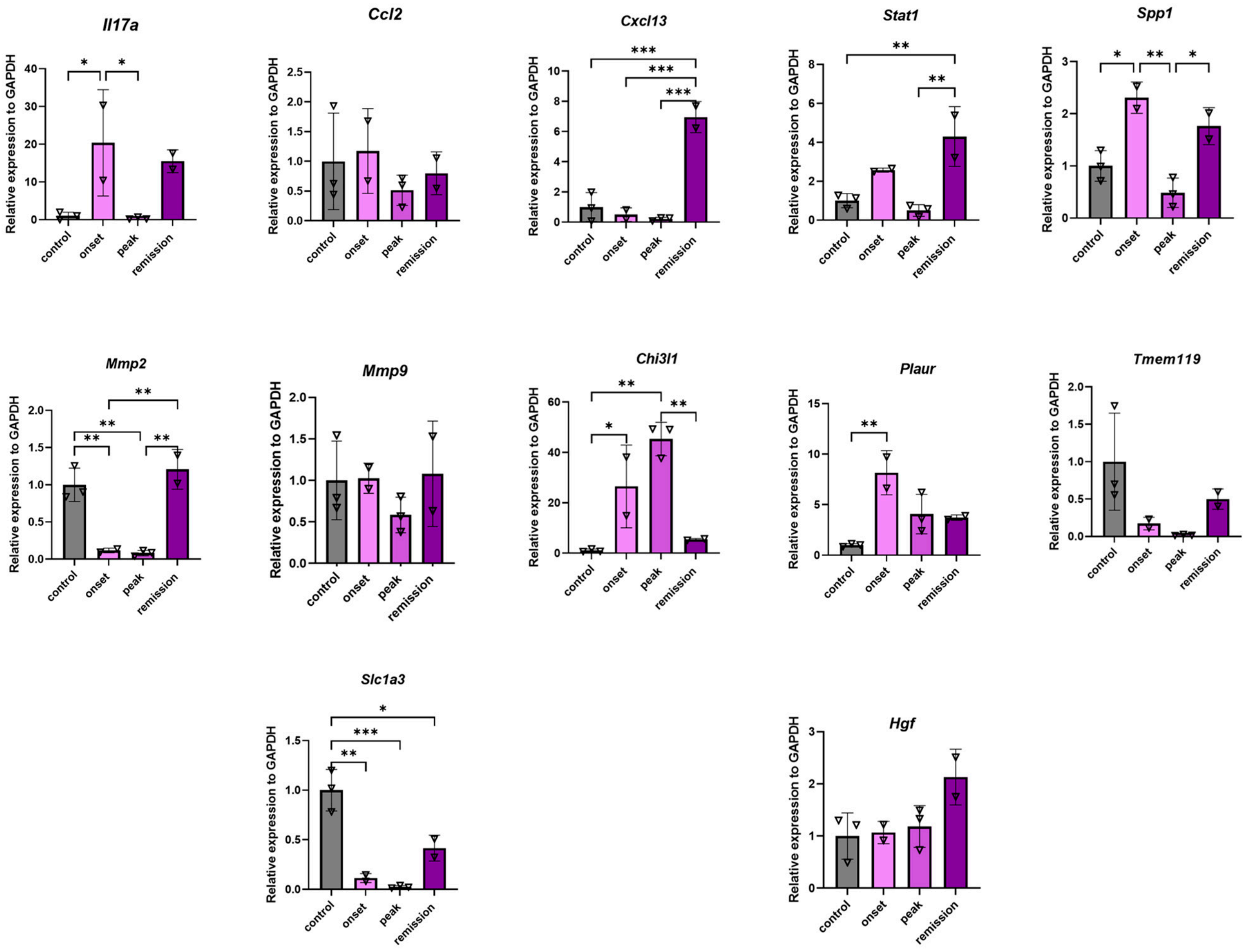

Supplementary Figure S2. Differential expression of genes in CD11b+ cells during the different phases of PLP-induced EAE. The levels of mRNA transcripts for the selected genes relative to Gapdh were measured in CD11b+ samples isolated from controls and EAE mice on the onset, peak and remission phase by RT-qPCR. A number of 2 to 3 mice was used in the experiment. Each symbol represents a sample. * $p < 0.05$, ** $p < 0.01$, *** $p < 0.001$, one-way ANOVA.
